# A randomized trial of brief intervention strategies in patients with alcohol-related facial trauma as a result of interpersonal violence

**DOI:** 10.1186/1940-0640-7-S1-A66

**Published:** 2012-10-09

**Authors:** Christine Goodall, Adrian Bowman, Iain Smith, Alex Crawford, Lisa Collin, Ian Holland, Andrew Carton, Fiona Oakey, Ashraf Ayoub

**Affiliations:** 1 Department of Dentistry, Glasgow University, Glasgow, Scotland, UK; 2 School of Mathematics & Statistics, University of Glasgow, Glasgow, Scotland, UK; 3 Child & Adolescent Psychiatry, Gartnavel Royal Hospital, Glasgow, Scotland, UK; 4 Renfrewshire Council for Alcohol, Renfrewshire, Scotland, UK; 5 Department of Addiction Psychiatry, Kershaw Unit, Gartnavel Royal Hospital, Glasgow, Scotland, UK; 6 Department of Oral and Maxillofacial Surgery,Falkirk & District Royal Infirmary, Falkirk, Scotland, UK; 7 Department of Oral and Maxillofacial Surgery, Monklands Hospital, Airdrie, Scotland, UK

## 

Facial trauma is associated with male gender, low socioeconomic status, alcohol misuse, and violence. Brief intervention (BI) for alcohol is effective at reducing consumption in patients presenting with facial trauma. Single-session control of violence for angry impulsive drinkers (SS-COVAID) is a new intervention that attempts to address alcohol-related violence. This study assessed the effect of SS-COVAID and BI on drinking and aggression in facial trauma patients. Male facial trauma patients who sustained their injuries as a result of interpersonal violence while drinking and who had Alcohol Use Disorders Identification Test (AUDIT) scores of ≥8 were randomized to either BI or SS-COVAID. Patients were followed up at six and 12 months, and drinking and aggression outcomes were analyzed. One hundred ninety-nine patients entered the trial, and 187 were included in the analysis. Of these, 165 (89%) considered themselves to be victims, 92 (51%) had sustained a previous alcohol-related injury, and 28 (15%) had previous convictions for violence. Both interventions resulted in a significant decrease in negative drinking outcomes over 12 months of follow-up (p < 0.001). Neither intervention had a significant effect on aggression scores, nor was there a significant difference between interventions in terms of either outcome. Both SS-COVAID and BI had a significant effect on drinking variables in this patient cohort. No effect on aggression was seen despite the fact that SS-COVAID specifically addresses the relationship between alcohol and violence. One reason for this may be that the facial trauma patients in this study considered themselves to be victims rather than aggressors. Another possibility is that, while BI may successfully address lifestyle factors such as hazardous or harmful drinking, it may not be effective in modifying personality traits such as aggression.

